# Estimating local protein model quality: prospects for molecular replacement

**DOI:** 10.1107/S2059798320000972

**Published:** 2020-03-03

**Authors:** Björn Wallner

**Affiliations:** a Division of Bioinformatics, Department of Physics, Chemistry and Biology, Linköping University, SE-581 83 Linköping, Sweden

**Keywords:** molecular replacement, protein model quality assessment, local error estimates, *ProQ*3*D*

## Abstract

Model quality assessment programs estimate the quality of protein models and can be used to estimate local error in protein models. *ProQ*3*D* is the most recent and most accurate version of our software. Here, it is demonstrated that it is possible to use local error estimates to substantially increase the quality of the models for molecular replacement (MR). Adjusting the *B* factors using *ProQ*3*D* improved the log-likelihood gain (LLG) score by over 50% on average, resulting in significantly more successful models in MR compared with not using error estimates. On a data set of 431 homology models to address difficult MR targets, models with error estimates from *ProQ*3*D* received an LLG of >50 for almost half of the models 209/431 (48.5%), compared with 175/431 (40.6%) for the previous version, *ProQ*2, and only 74/431 (17.2%) for models with no error estimates, clearly demonstrating the added value of using error estimates to enable MR for more targets. *ProQ*3*D* is available from http://proq3.bioinfo.se/ both as a server and as a standalone download.

## Abstract 2

Local error estimates can be used to improve the success of models in molecular replacement.

## 1. Introduction   

The estimation of protein model quality has a long history in protein structure prediction, originating from methods that estimate the free energy of protein models (Hendlich *et al.*, 1990 ▸; Jones *et al.*, 1992 ▸; Lüthy *et al.*, 1992 ▸). If the free energy of a protein can be accurately described, it should be possible to use this to find the minimum free energy and locate the native structure of the protein. However, the vast majority of energy functions describing the free energy have focused on identifying the native structure among a set of decoys (Park & Levitt, 1996 ▸). These methods do not necessarily show a good correlation with the relative quality of protein models, in particular for difficult homology modelling or *ab initio* cases.

In 2003, we developed the *ProQ* method, which had a different aim to previous methods (Wallner & Elofsson, 2003 ▸). Instead of recognizing the native structure among a set of decoys, *ProQ* was developed to predict the quality of a model using machine learning and features that could be calculated from the model itself, such as different types of atom–atom contacts, residue–residue contacts, surface-exposure preference, agreement with predicted secondary structure and surface area. We used *ProQ* to rank models in CASP5 and it was the main reason why our prediction servers were ranked at the very top in terms of model quality (Wallner *et al.*, 2003 ▸).

*ProQ* was later extended to estimate the local quality of each residue in a protein model, and the quality of the entire model was estimated by simply summing up the quality for each residue (Wallner & Elofsson, 2006 ▸). This method was rather successful in CASP7 (Wallner & Elofsson, 2007 ▸) and CASP8 (Larsson *et al.*, 2009 ▸), in which quality assessment had now become a separate prediction category.

In *ProQ*2, improved prediction was achieved by using evolutionary sequence profile weights and features averaged over the whole model, even though the prediction was local (Ray *et al.*, 2012 ▸; Uziela & Wallner, 2016 ▸). *ProQ*2 error estimates encoded as *B* factors were shown to improve the success of molecular replacement (MR) (Bunkóczi *et al.*, 2015 ▸). This was based on the idea that the estimation of local model quality could be translated into coordinate uncertainty and used to smear the atoms in the model over their range of possible positions (Read & Chavali, 2007 ▸), which was first implemented using ensemble consensus to estimate local errors (Pawlowski & Bujnicki, 2012 ▸).

Since the release of *ProQ*2, we have made considerable improvements in prediction accuracy. In *ProQ*3, we combined *ProQ*2 with two novel predictors based on centroid and all-atom energy terms calculated using *Rosetta* (Leaver-Fay *et al.*, 2011 ▸). Most recently, we developed *ProQ*2*D* and *ProQ*3*D* (Uziela *et al.*, 2017 ▸), which are deep-learning versions of *ProQ*2 and *ProQ*3 optimized on a larger training set using new developments in machine learning. In terms of performance, we have gradually improved Pearson’s correlation between predicted and actual quality from 0.60 for *ProQ* to 0.81 for *ProQ*2, 0.85 for *ProQ*2*D* and *ProQ*3, and finally 0.9 for *ProQ*3*D* calculated on data from CASP11 (Uziela *et al.*, 2017 ▸).

Given the recent improvements in prediction accuracy in *ProQ*3*D*, we wanted to analyze how this improvement propagates to the ability to improve the quality of the models for MR.

## 2. Methods   

### 2.1. Data set   

The data set consisted of 431 target–template pairs for 229 molecular-replacement targets with an LLG of <100, using the template to calculate the LLG, and resolution between 0.8 and 3.1 Å (see the supporting information for a complete list). The pairs have an average sequence identity of 28% (with a range of 17–45%) calculated using the alignment constructed below. Models for the pairs were constructed by first generating hidden Markov models (HMMs) for the target and template sequences, respectively, using *HHblits* (Remmert *et al.*, 2012 ▸) with two iterations against uniclust30_2018_08. The two HMMs for targets and template were then aligned using *HHalign* (Steinegger *et al.*, 2019 ▸) with default settings. 3D models were constructed from the alignment using *Modeller* version 9.14 (Šali & Blundell, 1993 ▸). In the default setting, N- and C-terminal regions unaligned with the template are trimmed from the model, but all other unaligned regions are kept.

### 2.2. Local error estimation   

Local errors were estimated using *ProQ*2 (Ray *et al.*, 2012 ▸) and *ProQ*3*D* (Uziela *et al.*, 2017 ▸). Both programs predict the *S* score (Cristobal *et al.*, 2001 ▸), a score between 0 and 1, where 0 is no quality and 1 is perfect quality. The score *S_i_* transforms the local distance deviation *d_i_* using the formula *S_i_*(*d_i_*) = 1/[1 + (*d_i_*/*d*_0_)^2^], where *d*_0_ is a parameter that monitors how fast the function goes to zero; here, *d*_0_ = 3.0 Å was used, which makes the transform most sensitive to distances around 3 Å; for example, the 0–6 Å range is mapped to [0.2–1], while all distances larger than 6 Å are mapped to [0–0.2]. The predicted local qualities *S_i_* were transformed to predicted local error estimates by solving the equation for *d_i_*: *d_i_* = *d*_0_(1/*S_i_* − 1)^1/2^. To restrict the range of *d_i_*, all *d_i_* > 15 were set to 15.

### 2.3. Molecular replacement   

To estimate the usefulness of models for molecular replacement, the log-likelihood gain (LLG) measure from *Phaser* (McCoy *et al.*, 2007 ▸) was used. The LLG measures how much better an atomistic model explains the measured X-ray data compared with a random model (Read, 2001 ▸). In the general case, calculating the LLG is time-consuming. However, for the purpose of this study we can utilize the fact that the target structures are available and can be used to place the models in roughly the optimal position by superimposing them on the target structures using *phenix.superimpose_pdbs* (Liebschner *et al.*, 2019 ▸). This faster version of *Phaser* (McCoy *et al.*, 2007 ▸) was used to calculate the LLG both without and with local error estimates from *ProQ*2 and *ProQ*3*D*.

To be able to compare different LLG values and their usefulness, an LLG of >50, corresponding to a 90% chance of success in MR (McCoy *et al.*, 2017 ▸), was used as threshold to define models of good quality for MR.

## 3. Results and discussion   

**Figure 1:**
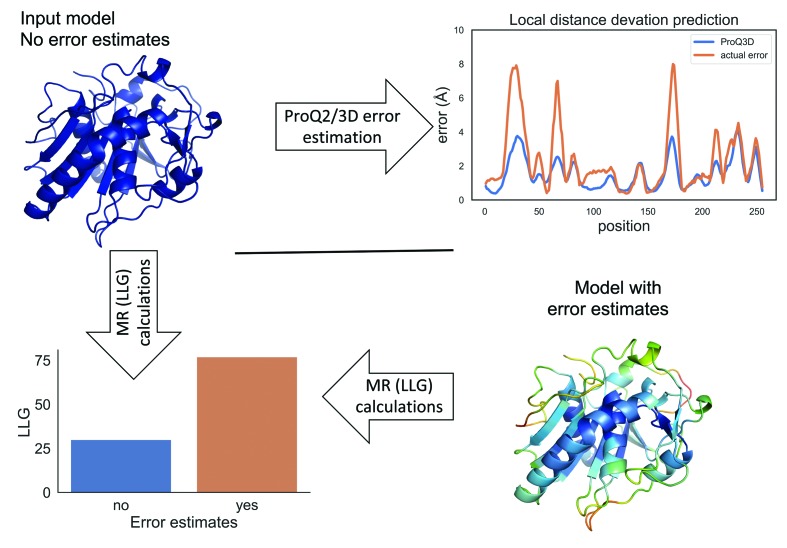
Overview of the workflow used. Input models superimposed on the target structures are used as input. Errors in the models are estimated using *ProQ*2 or *ProQ*3*D*; the errors are added to the *B*-factor column in the model. Models with both no error estimates and error estimates are used in MR calculations to estimate the LLG.

We wanted to compare the potential success in molecular replacement (MR) for the models in the data set (see Section 2) using *ProQ*2, *ProQ*3*D* and no error estimates. As outlined in the flowchart in Fig. 1 ▸, we first ran *Phaser* (McCoy *et al.*, 2007 ▸) on the models without any error estimates to establish a baseline. We then used *ProQ*2 and *ProQ*3*D* to predict residue-specific error estimates, as illustrated in the top right panel in Fig. 1 ▸, and added these to the *B*-factor column of the model (see the model colored by predicted error in the bottom right panel in Fig. 1 ▸). Finally, *Phaser* was run again with the same model, but now with error estimates. Following this procedure, three LLG values were calculated for each of the 431 models in the data set: without error estimates, with *ProQ*2 error estimates and with *ProQ*3*D* error estimates, respectively.

### 3.1. Model quality in MR   

**Figure 2:**
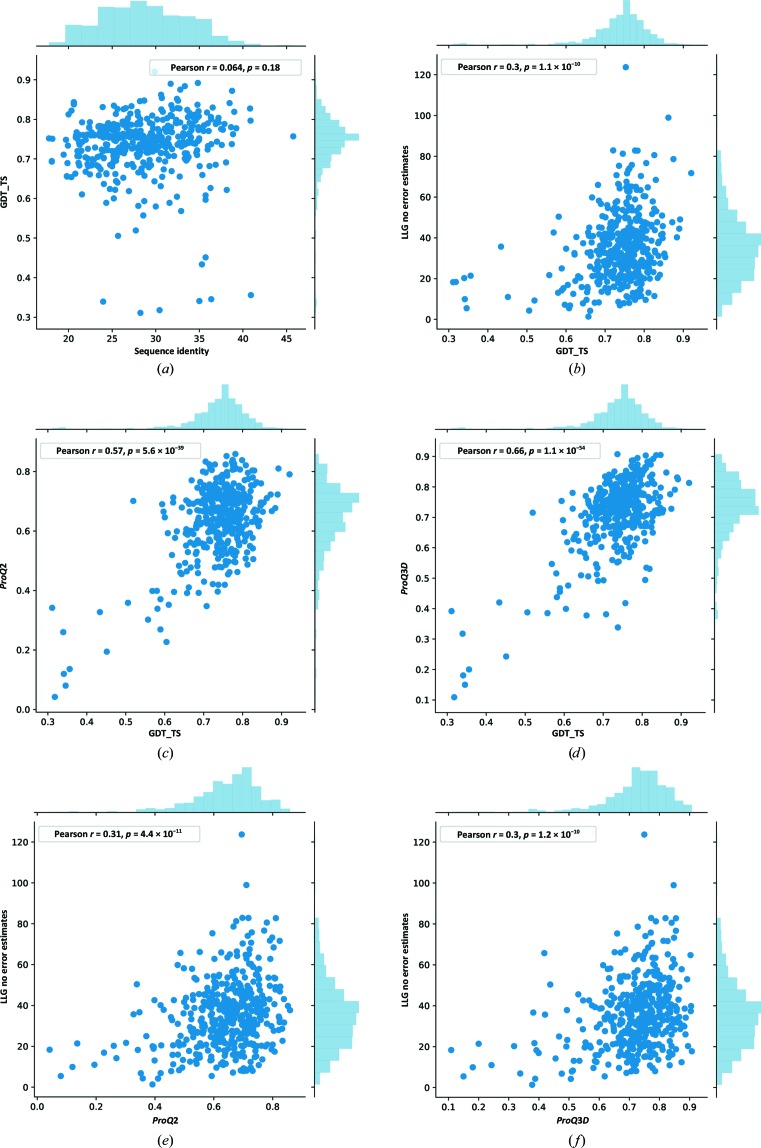
Global model quality and potential success in MR. (*a*) Sequence identity for the target–template sequences versus global model quality measured by GDT_TS, (*b*) GDT_TS against the LLG for a model without error estimates, (*c*) GDT_TS in relation to the predicted global quality by *ProQ*2, (*d*) GDT_TS in relation to the predicted global quality by *ProQ*3*D*, (*e*) *ProQ*2 against the LLG for a model without error estimates, (*f*) *ProQ*3*D* against the LLG for a model without error estimates.

The target sequences from all models have a relatively low sequence identity to the templates, with a majority (61%) below 30%; however, the produced models are still relatively accurate overall, with most GDT_TS (Zemla, 2003 ▸) values above 0.7, corresponding to roughly 70% correct residues (Fig. 2 ▸ *a*). It can also be noted that at this sequence-identity level there is almost no correlation (0.06) between the sequence identity and the quality of the models. Next, we analyzed whether the quality of the models (GDT_TS) is important for the models to be useful in MR as measured by the LLG for the models without error estimates (Fig. 2 ▸ *b*). Indeed, models with high LLG are also of high quality, and almost all cases (LLG > 50) have GDT_TS > 0.7. However, not all high-quality models receive a high LLG. In fact, quite a few models with GDT_TS above 0.7 have an LLG of less than 50. Thus, it is not only the overall quality of the model that impacts on whether a model is of good quality for MR.

Both *ProQ*2 and *ProQ*3*D* predict global overall model quality based on its local error estimates. The correlation to the correct GDT_TS measure in this data set is 0.57 and 0.66 for *ProQ*2 and *ProQ*3*D*, respectively (Figs. 2 ▸ *c* and 2 ▸ *d*). As we know from previous experience, both *ProQ*2 and *ProQ*3*D* are very good at separating bad from good models, but not as good when it comes to ranking already high-quality models. In this case, both *ProQ*2 and *ProQ*3*D* are able to discriminate between low-quality and high-quality models, and almost all cases with LLG > 50 have a *ProQ* score above 0.5 (Figs. 2 ▸ *e* and 2 ▸ *f*). In addition, the relation between *ProQ*2 and *ProQ*3*D* to LLG is very similar to the relation between GDT_TS and LLG (compare Figs. 2 ▸ *e* and 2 ▸ *f* with Fig. 2 ▸ *b*). Thus, it should be possible to use a threshold on the *ProQ* score to predict whether a model is of good quality for MR.

### 3.2. MR with error estimates   

**Figure 3:**
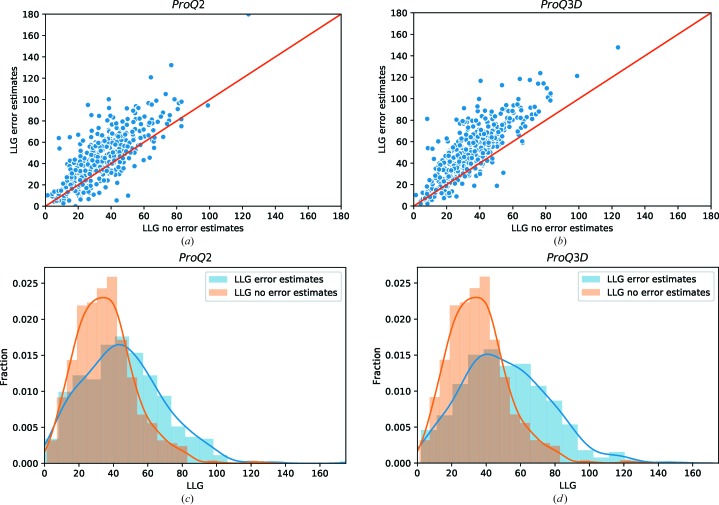
LLG values without error estimates and with error estimates from *ProQ*2 and *ProQ*3*D*.

Next, we calculated the LLG using models with error estimates from *ProQ*2 and *ProQ*3*D* (Fig. 3 ▸). Clearly, for the vast majority of the models *ProQ*2 and *ProQ*3*D* error estimates improve the LLG compared with no error estimates (Figs. 3 ▸ *a* and 3 ▸ *b*). *ProQ*3*D* improves 383/431 (88.9%) of the models, which is significantly larger than the 329/431 (76.3%) of the models that were improved by *ProQ*2 (Table 1 ▸).

We can also observe a clear shift in the LLG distribution towards higher LLG values when using error estimates (Figs. 3 ▸ *c* and 3 ▸ *d*). For *ProQ*3*D* the average LLG increases from 〈LLG_noerror_〉 = 35.8 to 〈LLG_error_〉 = 51.7. In terms of modelling there is a small advantage to pruning all unaligned regions from the search model when not using error estimates, 〈LLG_noerror-pruned_〉 = 36.5 (an increase of 0.7), and a small disadvantage when using error estimates, 〈LLG_error-pruned_〉 = 51.4 (a decrease of 0.3). In both cases, the advantage of using error estimates is clear.

In a previous study, we reported an average 25% increase in the LLG using *ProQ*2 error estimates compared with models using no error on models submitted to CASP10 (Bunkóczi *et al.*, 2015 ▸). Here, the average improvement in the LLG using *ProQ*2 is 36.7% (Table 1 ▸); since there is no change in method­ology between the two sets, this number indicates that this particular data set is slightly easier than the CASP10 data set. *ProQ*3*D* error estimates improve the average LLG by 52%, suggesting that the success in MR can be improved even further by using *ProQ*3*D* instead of *ProQ*2. Indeed, if we check how many models that have LLG values indicating a high chance of success (LLG > 50), we see that only 74/431 models without error estimates are successful, while 175/431 and 209/431 are successful using *ProQ*2 and *ProQ*3*D*, respectively; the difference between *ProQ*2 and *ProQ*3*D* is significant.

### 3.3. Prediction example   

**Figure 4:**
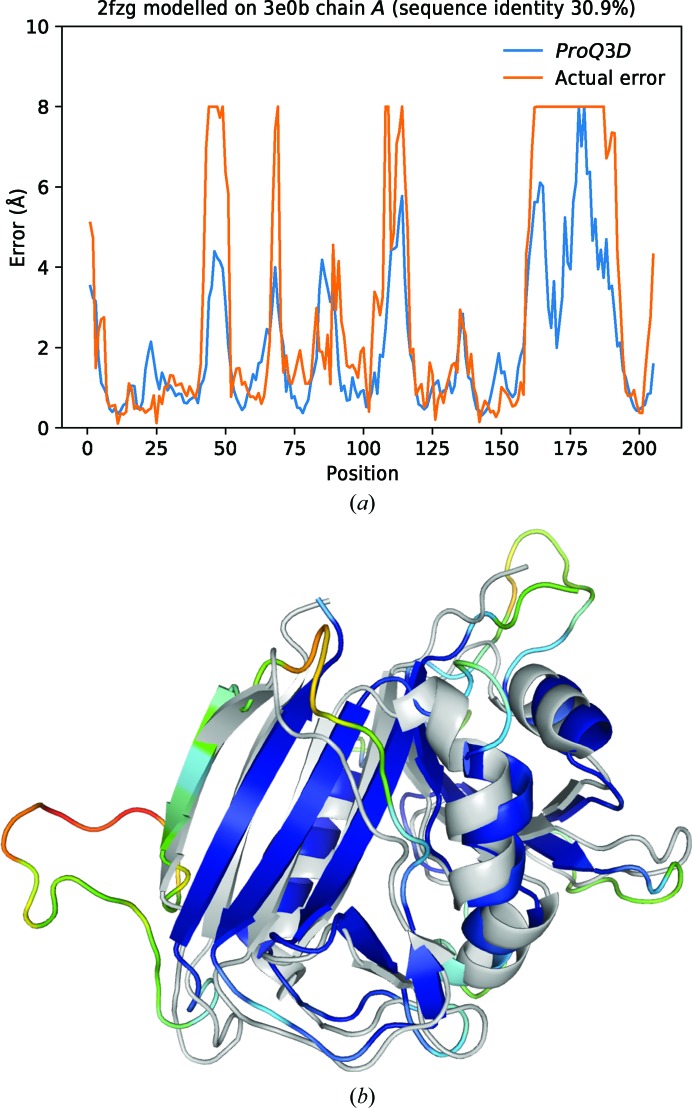
Prediction example for the target PDB entry 2fzh modelled on PDB entry 3e0b chain *A*. (*a*) The predicted error estimates by *ProQ*3*D* compared with the actual error. (*b*) The model colored by *ProQ*3*D*-predicted error and superimposed on the template (grey) used to build the model.

Finally, we conclude by demonstrating a successful prediction case. The target is a 206-amino-acid dihydrofolate reductase from *Pneumocystis carinii* solved using X-ray diffraction at 2.1 Å resolution (PDB entry 2fzh). The template is a 332-amino-acid dihydrofolate reductase from *Bacillus anthracis* solved using X-ray diffraction at 2.25 Å resolution (PDB entry 3e0b, chain *A*). The alignment between the target and template sequence is 30.9% identical and the quality of the model based on this alignment has a GDT_TS of 0.67. The predicted error by *ProQ*3*D* as well as the actual error (capped at 8 Å) is shown in Fig. 4 ▸(*a*). The correlation between the predicted and actual error is 0.85. The model colored by the error with the corresponding template superimposed is shown in Fig. 4 ▸(*b*); some obvious bad loops that do not align well with the template are correctly identified as such, but then there are also some secondary-structure elements, such as the leftmost strand, which align well with the template but are correctly predicted as bad (data not shown). The model without error estimates received an LLG of 8.2 and this improved to 81.3 for the model with error estimates, clearly demonstrating the value of using error estimates.

## 4. Conclusion   

We have demonstrated that the use of error estimates can increase the number of models useful for MR substantially. The most recent version of our model-quality assessment program *ProQ*3*D* is more accurate and significantly better than *ProQ*2. *ProQ*3*D* improved the LLG score by over 50% on average, resulting in significantly more models of good quality for MR compared with not using error estimates. *ProQ*3*D* is available from http://proq3.bioinfo.se/ both as a server and as a standalone download.

## Supplementary Material

Models and local quality estimates. DOI: 10.1107/S2059798320000972/rr5193sup1.zip

Description of data set. DOI: 10.1107/S2059798320000972/rr5193sup2.xlsx

## Figures and Tables

**Table 1:** LLG improvements using error estimates for 431 models in the data set LLG increase is the fractional improvement in LLG when using error estimates, #targets ΔLLG>0 is the number of targets that improve when using error estimates and #targets LLG>50 is the number of targets that have an LLG of >50.

Method	LLG increase (%)	#targets ΔLLG>0	#targets LLG>50†
No error	0.0	0 (0.0%)	74 (17.2%)
*ProQ*2	36.7	329 (76.3%)	175 (40.6%)‡
*ProQ*3*D*	52.0	383 (88.9%)§	209 (48.5%)¶
True errors	116.9	425 (98.6%)	318 (73.8%)

† Corresponding to 90% chance of success in MR (McCoy *et al.*, 2017 ▸).

‡ *ProQ*2 significantly better than no error on LLG > 50 (*p* < 10^−21^, binomial test).

§ *ProQ*3*D* significantly better than *ProQ*2 on ΔLLG > 0 (*p* < 10^−10^, binomial test).

¶ *ProQ*3*D* significantly better than *ProQ*2 on LLG > 50 (*p* < 10^−3^, binomial test).
